# Viruses and Cancer: An Accidental Journey

**DOI:** 10.1371/journal.ppat.1005573

**Published:** 2016-09-08

**Authors:** Karl Munger

**Affiliations:** Department of Developmental, Molecular & Chemical Biology, Tufts University School of Medicine, Boston, Massachusetts, United States of America; The Fox Chase Cancer Center, UNITED STATES

Infections with “high-risk” mucosal human papillomaviruses (HPVs) cause approximately 5% of all cancers worldwide. These include almost all cases of cervical carcinoma (a leading cause of cancer death in women), a large fraction of other anogenital tract cancers, and oropharyngeal tumors. For the last 30 years, my research has been focused on the molecular mechanisms by which HPVs contribute to cancer formation. In an article published some years ago, I posed the question of whether such behavior “represents relentless scientific curiosity or is simply a reflection of intellectual lethargy”. This short article attempts to convince readers that it is the former rather than the latter possibility.

As a biochemistry PhD student at the University of Zürich, Switzerland, I had studied copper and zinc homeostasis in *Neurospora crassa*. My amazing mentor, Konrad Lerch, almost instantly infused me with his boundless enthusiasm for all things research. My interest in cancer biology began when I came across Bob Weinberg’s remarkable *Nature* paper, in which they identified RAS as a major transforming entity in a human bladder carcinoma line. Astoundingly, the RAS mutant they isolated from the cancer line was identical to a previously identified mutant RAS carried by an oncogenic retrovirus. When I started out as a postdoc in Peter Howley’s group at the National Institutes of Health in Bethesda, Maryland, it had just been determined that E6 and E7 were the only HPV proteins consistently expressed in cervical carcinoma lines. Like the metallothioneins that I studied for my PhD thesis, papillomavirus E6 and E7 are low molecular size, cysteine-rich, zinc-binding proteins that lack intrinsic enzymatic activities. The big question was whether E6 and E7 were carcinogenic drivers or merely innocent passengers. These were exhilarating times, and since access to the NIH campus did not yet involve airport-style security, there was action in the lab at all times, day or night. We discovered that E7 and E6 indeed were carcinogens and that they contributed to oncogenesis at least in part by targeting the cellular retinoblastoma and p53 tumor suppressors, respectively. A particularly formative experience was an annual “no holds barred,” open notebook lab meeting of members of the Livingston, Weinberg, Harlow, and Howley groups that was initially focused on studies involving the retinoblastoma tumor suppressor. The conference was held in a historic farm in Colrain, a bucolic town in Western Massachusetts. In addition to amazing science, it featured equally amazing food prepared and served by master chefs David Livingston and Bob Weinberg. Copious amounts of adult beverages kept the conversations going. I learned that even though competition drives scientific progress, research should not be a bloodsport, and in general it is more productive to solve problems with help from your friends. Many of my NIH and Colrain colleagues have indeed remained trusted friends and collaborators to this day.

Lacking any artistic talents, my creed that basic researchers have much in common with artists may be mostly self-serving. But similar to artists, the significance and usefulness of our achievements is often not immediately obvious and appreciated. Breakthrough technologies such as the polymerase chain reaction (PCR), RNA interference, or CRISPR/CAS9–based genome editing were enabled by basic research on what some may refer to as obscure processes in obscure organisms. Without a robust pipeline that is fueled by basic science, clinical translation will quickly boil down to continuous retranslation of worn-out concepts with occasional semantic repackaging to make them appear fresh.

It is ironic that at this time of almost unlimited experimental opportunities, basic researchers are to face major, potentially existential challenges. Many are caused by the rapidly diminishing funds for basic research and are exacerbated by a seemingly unstoppable avalanche of rules and regulations that codify every conceivable aspect of research. These developments have the potential to drive worrisome academic climate change. An emerging mantra is that research is to be run like a business: strictly focused on quantifiable, short term outcomes. Accordingly, faculty support and job security are mere obstacles to innovation; researchers ought to be nimble, and, like the proverbial lemmings, they are expected to follow today’s most grant dollar- and publicity-generating scientific fads.

Only if this climate change can be halted will basic biomedical research continue to attract the bright and energetic individuals who will make the real breakthroughs. Viral oncology remains an area ripe for such discoveries. The elegant strategies that these viruses have evolved to reprogram key cellular signaling circuits are amazing. As I first came to realize when reading Weinberg’s RAS paper, virally targeted pathways are frequently dysfunctional in cancers not caused by viruses.

It is remarkable that incredibly efficacious prophylactic vaccines that can prevent the majority of disease and cancer caused by mucosal HPV infections have been brought to market within less than 25 years of the first report of HPV16 detection in human cervical cancers. There cannot be a better testament to the power and importance of basic biomedical research.

Lastly, in these times, where it is fashionable for “outsiders” to question even the most basic scientific and academic concepts, it is useful to remember James Madison, who wrote that “…the advancement and diffusion of knowledge, which is the only guardian of true liberty, the great cause to which [his] life has been devoted.” While I am not Thomas Jefferson (to whom Madison refers to), I feel privileged to call basic research my passion.

**Image 1:**
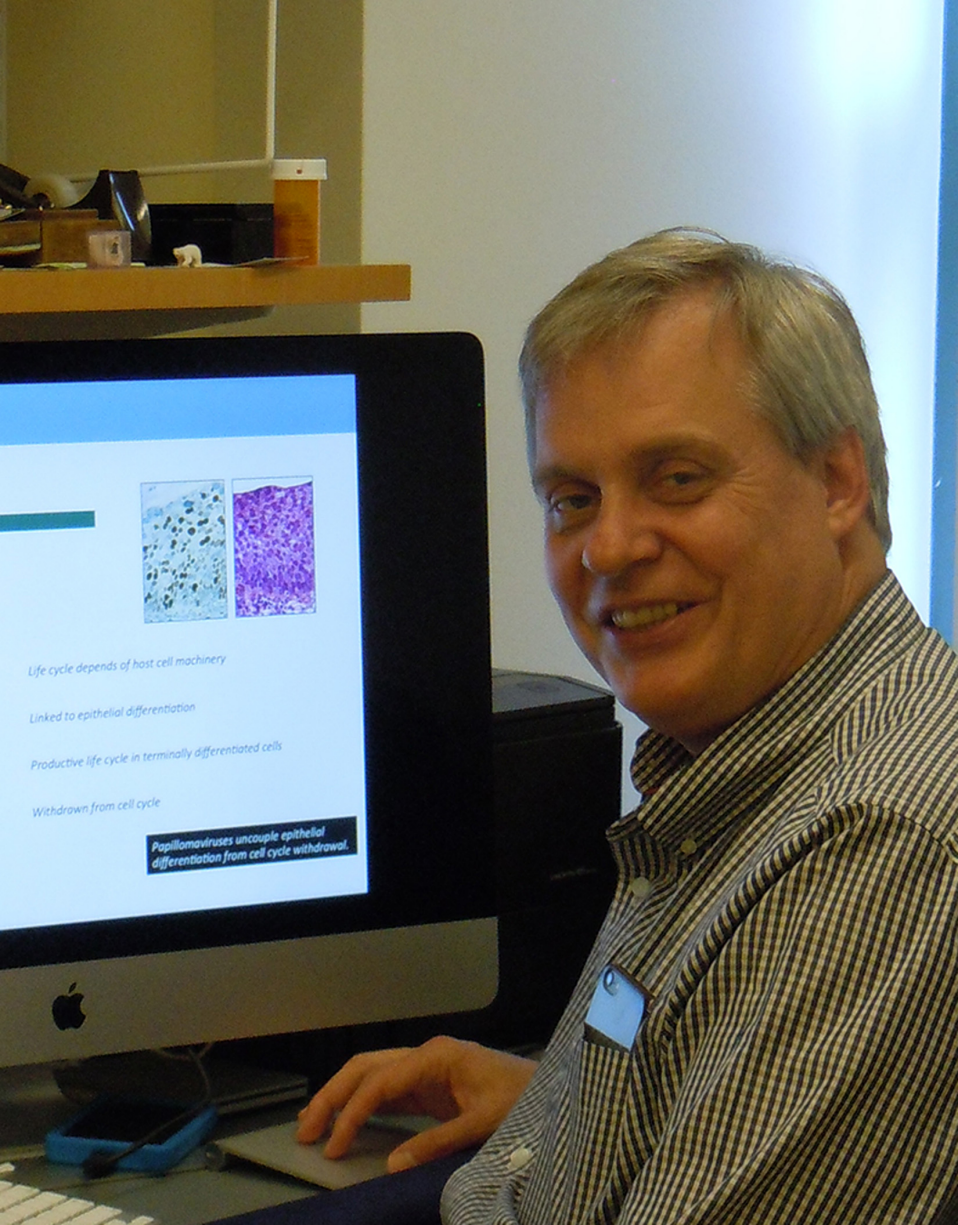
Karl Munger.

